# A New Type of Ultrasonic Gyroscopic Sensor Based on a Solid-State Standing-Wave Vibrator: Towards Shock-Resistant Design

**DOI:** 10.3390/s26092798

**Published:** 2026-04-30

**Authors:** Michail Shevelko, Andrey Baranov, Ekaterina Popkova, Yasemin Staroverova, Alexander Kukaev, Sergey Shevchenko

**Affiliations:** 1 Department of Electroacoustics and Ultrasonic Technology, Saint Petersburg Electrotechnical University, 197022 St. Petersburg, Russia; mmshevelko@etu.ru (M.S.); adbaranov@etu.ru (A.B.); yadurukan@etu.ru (Y.S.); 2 Department of Laser Measurement and Navigation Systems, Saint Petersburg Electrotechnical University, 197022 St. Petersburg, Russia; askukaev@etu.ru (A.K.); syshevchenko@etu.ru (S.S.)

**Keywords:** ultrasonic gyroscopic sensor, shock-resistant sensitive element, standing acoustic wave, solid-state vibrator

## Abstract

**Highlights:**

**What are the main findings?**

- A new type of ultrasonic gyroscopic sensor—a solid-state standing-wave vibrator is developed.
- The level of the informative signal of the new sensor on standing waves significantly exceeds the signal level of similar solutions on traveling waves.

**What are the implications of the main findings?**

- A novel sensor design concept overcomes the limitations of MEMS sensors due to the absence of inertial masses.
- The developed solid-state standing-wave vibrator is expected to be shock-resistant due to the absence of inertial masses.

**Abstract:**

This paper presents a new type of ultrasonic gyroscopic sensor based on a solid-state standing-wave vibrator, which is promising for shock-resistant applications. A theoretical model of the proposed design, which is a layered structure, and the numerical simulation of its frequency response using the developed software are presented. A test sample of the novel sensing element was made and experimental studies of its frequency response were conducted. The results showed a high correlation between the resonant frequencies both for the real sample research and numerical modeling; thus, the validity of the theoretical model was confirmed. The laboratory investigation of the developed sensing element on a test bench under rotating conditions was carried out and a shift in the standing-wave amplitude proportional to the angular velocity of rotation was revealed; thus, an informative signal for this type of gyroscopic sensor was found. It is shown that the amplitude of the output signal of the new sensor on standing waves compares favorably with the signal levels reported for similar traveling-wave solutions in previous studies. The optimization strategies for the new sensor’s design and operating mode to increase signal to noise ratio are also identified. Thus, the potential of using the developed solid-state standing-wave vibrator as a shock-resistant ultrasonic gyroscopic sensor is supported.

## 1. Introduction

Modern inertial navigation systems require angular rate sensors to combine high accuracy, compactness, reliability, and robustness. Over the past decades, several fundamentally different approaches to gyroscope implementation have been proposed and developed, each with its own advantages and limitations:

1. Microelectromechanical (MEMS) gyroscopes are the dominant solution in consumer electronics, automotive and other applications where cost and size are important [1,2,3,4]. They operate based on the Coriolis effect: an oscillating mass experiences a transverse force proportional to the angular velocity as the system rotates. Despite significant advances in micromachining technologies, which have allowed high precision and stability to be achieved even at a level sufficient for tactical applications, a key drawback of MEMS devices remains their sensitivity to external mechanical influences due to the presence of moving parts connected to the frame through thin elastic structures, which leads to degradation of performance under strong vibration and shock up to their complete failure; in addition, even minor residual stresses or frame deformations during thermal cycling can cause significant zero drift and detuning of resonant frequencies. This fundamental vulnerability limits the use of classical MEMS solutions in areas where maximum reliability is required [1].
2. Optical gyroscopes (OGs) are based on the Sagnac effect; their operation relies on detecting the phase or frequency difference between two counterpropagating light waves in a closed optical circuit during rotation of the system [5,6]. This class includes fiber optic gyroscopes (FOGs) and ring laser gyroscopes (RLGs).

RLGs are characterized by high sensitivity, a linear response characteristic, and resistance to external mechanical influences. However, they are affected by so-called “lock-in” at low rotation velocities, require complex compensation measures and are large in size and power consumption.

FOGs, in contrast, use a passive optical coil made of multi-turn optical fiber through which two incoherent or coherent light beams from an external source are passed. Rotation causes a phase shift between them, which is detected interferometrically. FOGs are characterized by exceptional reliability, a long service life, and the ability to increase accuracy by increasing fiber length.

Despite their high metrological characteristics, both types of optical gyroscopes are limited in application due to their relatively large size, high cost and manufacturing complexity, making them inapplicable for mass-produced, compact systems where more miniaturized solutions are preferred.

3. Wave solid-state gyroscopes (WSGs) offer an intermediate solution between MEMS and OGs. This class includes both gyroscopes based on the oscillations of macroscopic resonators and devices using surface and bulk acoustic waves [7,8,9]. Their operating principle also relies on the inertial effect of the precession of a standing elastic wave in an axisymmetric resonator during its rotation. This type of gyroscope has no wave propagation in the sense of space energy transfer: all the oscillatory energy is localized within the resonator and used to maintain the standing mode.

The most developed in this class are hemispherical resonator gyroscopes (HRGs). They demonstrate characteristics comparable to OGs, while having significantly smaller dimensions and weight. However, their production is associated with extremely complex and expensive quartz resonator balancing technologies, which limit their mass application. Gyroscopes based on ring and cylindrical resonators, manufactured using MEMS technology, attempt to overcome this barrier, but face the same challenges as classic MEMS devices: the influence of suspensions on the quality factor and sensitivity to external mechanical influences. This creates a need for new, more reliable physical principles, which are implemented in gyroscopes using acoustic waves.

4. Acoustic solid-state gyroscopes (ASGs) operate on the gyroscopic effect of elastic interaction between particles of a solid body during the propagation of surface (SAW) or bulk (BAW) acoustic waves and contain no moving masses or suspensions, which provides them with high shock and vibration resistance [10,11].

SAW gyroscopes are a class of solid-state inertial sensors that use elastic waves propagating near the surface of a piezoelectric crystal. Due to the absence of moving mechanical elements, such devices offer high shock and vibration resistance, small dimensions, low power consumption and easy manufacturing. Currently, three main concepts for constructing SAW-based angular velocity sensors can be distinguished [12,13,14,15,16].

The first concept is based on the dependence of the SAW phase velocity on the angular velocity of the medium. This effect was first theoretically described by B. Lao and consists of a change in the SAW velocity during rotation of a piezoelectric half-space proportional to the angular velocity and inversely proportional to the wave frequency as a shift in the resonant frequency of the SAW resonator or a phase change in the delay line [17]. This approach is implemented, for example, in circuits with two counterpropagating traveling waves, where the difference in their phases (or frequencies) is an informative signal [12].

The second concept uses the generation of a so-called secondary SAW under the influence of the Coriolis force [15,16]. In this circuit, the primary SAW is excited in one direction and forms a standing wave in the resonator. Particles of the medium at the antinodes of this wave perform elliptical oscillations, and during rotation, the resulting Coriolis force induces a secondary SAW, propagating orthogonally to the primary one. To amplify the signal, a micro mass array is placed in the antinode zone of the standing wave, increasing the effective mass and, consequently, the magnitude of the Coriolis force. The secondary wave is detected by a separate interdigital transducer (IDT), with the output signal amplitude proportional to the angular velocity. The disadvantage of this concept is the low level of the informative signal and the need to suppress parasitic acoustic interference from the primary wave.

The third, more modern concept involves the use of crystallographic directions in which SAW propagation is not accompanied by the generation of an electric field—so-called non-piezoelectric directions [17]. In the absence of rotation, there is no output signal at the receiving IDT. However, during rotation, the symmetry is broken and the Coriolis force causes a redistribution of deformations, resulting in a piezoelectric response proportional to the angular velocity. This principle significantly reduces background acoustic interference and increases sensitivity.

Although SAW gyroscopes have become widespread in modern microelectromechanical systems due to their compatibility with planar technologies and miniaturization potential, BAW-based devices represent an equally promising approach due to BAW propagation within the bulk of the material [18] rather than in the near-surface layer and, therefore, significantly lower susceptibility to the external mechanical deformations, temperature gradients and surface contaminants. Thus, BAW sensors demonstrate increased stability, reliability and resilience to harsh operating conditions.

The authors research team from the Department of Electroacoustics and Ultrasonic Technology at the Saint-Petersburg Electrotechnical University ETU “LETI” has developed a number of concepts for the sensitive element of ASGs. These concepts are based on various physical effects of the inertial interaction of elastic waves in a rotating medium, with different approaches to detect the informative signal [19,20].

The first concept is based on the effect of rotation of the polarization plane of a linearly polarized shear BAW as it propagates along the axis of rotation of an acoustic duct: with direct recording of the orthogonal component, reflection from an inclined face and the transformed wave at the interface. This approach ensures spatial and polarization mode separation, which reduces the noise level.

The second concept uses the direct excitation of two circularly polarized waves by a specialized transducer, propagating in opposite directions. Their velocities change during rotation, resulting in the accumulation of a phase difference between them, directly proportional to the angular velocity. The differential phase method ensures high noise immunity.

The third concept is based on the phenomenon of wave-type conversion. A linearly polarized shear BAW is excited in the acoustic duct. As the duct rotates under the influence of the Coriolis force, the polarization vector of this wave is distorted, resulting in the appearance of an orthogonal longitudinal component. This longitudinal component is then detected by a receiving transducer sensitive to longitudinal deformations and its amplitude is proportional to the angular velocity of rotation.

In this paper the authors propose a new concept of a solid-state ultrasonic sensing element of an angular velocity sensor, which makes it possible to combine the advantages of the above-mentioned concepts to potentially improve gyroscopic sensitivity while maintaining the small dimensions of the structure and manufacturing technology.

## 2. Numerical Analysis

### 2.1. Theoretical Basis

In the existing concept of a BAW angular velocity sensor’s sensing element [19], the informative signal is the rotation of the shear wave’s polarization vector due to the Coriolis force, which is detected by the emitting and receiving piezoelectric plates oriented at 90°. The informative component, proportional to the angular velocity, accumulates along the acoustic path length, which precludes sufficient miniaturization of the sensing element’s design.

The new gyroscopic sensor concept discussed in the present paper solves this scientific and technical problem by amplifying a low informative signal by creating a standing wave in the acoustic duct. By matching the thicknesses of the structural elements of the sensor’s layered configuration, which are multiples of half the standing wave’s wavelength *n*∙λ/2, the system’s transfer coefficient is significantly increased due to optimal resonance conditions, resulting in maximum gain.

**Figure 1:**
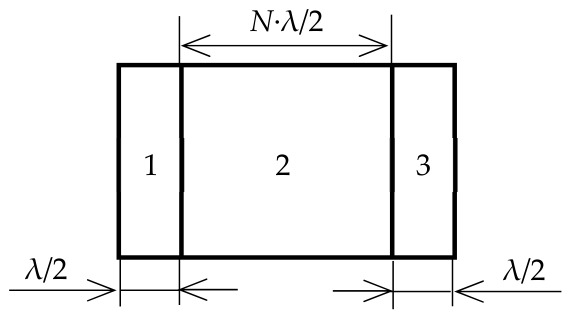
The new concept of solid-state acoustic gyro sensor design—the layered structure on standing waves (1—the emitting transducer; 2—the acoustic duct; 3—the receiving transducer).

The developed gyro-sensing element is a composite plate-type vibrator comprising a cylindrical acoustic duct made of an isotropic material with plate-type piezoelectric shear wave transducers mounted on its opposite ends, with the transducers’ sensitivity axes perpendicular to each other as shown in Figure 1.

The acoustic duct is made of a material with low sound absorption (in this case, glass) while its length d and the emitted signal duration τ satisfy the relations:

(1) $$\left\{\begin{array}{c} d=n\frac{\lambda }{2}=\frac{v_{t}n}{2f}\\ \tau \gg \frac{2D}{v_{t}} \end{array}\right.$$

where λ is the wavelength in the acoustic duct material at frequency *f*, $v_{t}$—the phase velocity of the shear wave in the material, *f*—the frequency of the excited oscillations, *n*—a natural number determining the order of the resonant mode. Fulfilling these conditions ensures the excitation and maintenance of standing shear waves in the acoustic duct. The resulting oscillations under rotation will have the form of a “twisted-shear” wave.

### 2.2. Theoretical Model

**Figure 2:**
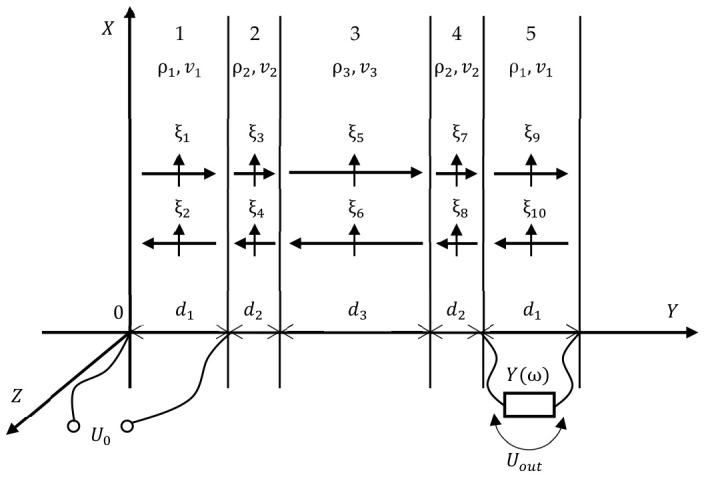
The theoretical model of the layered plate-type vibrator.

To mathematically describe the oscillatory system, we solved the problem of shear wave propagation in a one-dimensional approximation. The validity of using the classical one-dimensional modeling approach to this problem is due to the fact that the transverse size of the acoustic duct significantly exceeds the wavelength. The presented system consists of five successive layers: 1—the emitting transducer (piezoelectric material, *Y*-cut langasite); 2—the adhesive layer (epoxy resin); 3—the acoustic duct (fused quartz); 4—the adhesive layer (epoxy resin); 5—the receiving transducer (piezoelectric material, *Y*-cut langasite), as shown in Figure 2.

In this case, the coaxial orientation of the emitter and receiver sensitivity axes is investigated. Then, the rotation of the polarization vector of a shear wave can be considered equivalent to the shear wave propagating in the same direction, but with the orthogonal polarization direction (orthogonal to the direction of propagation as well).

The obtained result does not take into account the gyroscopic component of the transmission coefficient τΩ. The system’s transmission coefficient is modeled for the orthogonal component of the oscillations that arise as a result of the rotation due to the Coriolis force.

Since the system is resonant and contains standing waves, each standing wave in each layer is represented as a superposition of two waves traveling in opposite directions, which are characterized by their own amplitude. Thus, for the entire five-layer structure, it is necessary to determine 10 unknown amplitudes (two for each of the five layers):

(2) $$\xi_{1}=\xi_{1m}e^{-jk_{1}y}e^{j\omega t}\;;\;\xi_{2}=\xi_{2m}e^{jk_{1}y}e^{j\omega t}\;\;\xi_{3}=\xi_{3m}e^{-jk_{2}\left(y-d_{1}\right)}e^{j\omega t}\;;\;\xi_{4}=\xi_{4m}e^{jk_{2}(y-d_{1})}e^{j\omega t}\;\;\xi_{5}=\xi_{5m}e^{-jk_{3}\left(y-d_{1}-d_{2}\right)}e^{j\omega t}\;;\;\xi_{6}=\xi_{6m}e^{jk_{3}\left(y-d_{1}-d_{2}\right)}e^{j\omega t}\;\;\xi_{7}=\xi_{7m}e^{-jk_{2}\left(y-d_{1}-d_{2}-d_{3}\right)}e^{j\omega t}\;;\;\xi_{8}=\xi_{8m}e^{jk_{2}(y-d_{1}-d_{2}-d_{3})}e^{j\omega t}\;\;\xi_{9}=\xi_{9m}e^{-jk_{1}\left(y-d_{1}-d_{2}-d_{3}-d_{2}\right)}e^{j\omega t}\;;\;\xi_{10}=\xi_{10m}e^{jk_{1}(y-d_{1}-d_{2}-d_{3}-d_{2})}e^{j\omega t}\;,$$

where $\xi_{im}$ is the corresponding complex amplitude of the wave, $k_{q}=\omega /v_{t}$ is the wave number of the q^th^ layer, ω is the angular frequency, and $d_{q}$ is the thickness of the q^th^ layer.

To solve this problem, the boundary conditions are determined at the interlayer and external boundaries of the system. For the interlayer boundaries, the following conditions of continuity of oscillatory displacements are satisfied:

(3) $${\left.\xi_{1}+\xi_{2}\right|}_{y=d_{1}}={\left.\xi_{3}+\xi_{4}\right|}_{y=d_{1}}\;{\left.\xi_{3}+\xi_{4}\right|}_{y=d_{1}+d_{2}}={\left.\xi_{5}+\xi_{6}\right|}_{y=d_{1}+d_{2}}\;{\left.\xi_{5}+\xi_{6}\right|}_{y=d_{1}+d_{2}+d_{3}}={\left.\xi_{7}+\xi_{8}\right|}_{y=d_{1}+d_{2}+d_{3}}\;\;{\left.\xi_{7}+\xi_{8}\right|}_{y=d_{1}+d_{2}+d_{3}+d_{2}}={\left.\xi_{9}+\xi_{10}\right|}_{y=d_{1}+d_{2}+d_{3}+d_{2}}.$$

In addition, the condition of continuity of mechanical stresses is satisfied for all boundaries:

(4) $${\left.\sigma_{12}^{I}\right|}_{y=0}=0\;;{\left.\sigma_{12}^{I}\right|}_{y=d_{1}}={\left.\sigma_{12}^{II}\right|}_{y=d_{1}}\;{\left.\sigma_{12}^{II}\right|}_{y=d_{1}+d_{2}}={\left.\sigma_{12}^{III}\right|}_{y=d_{1}+d_{2}}\;;{\left.\sigma_{12}^{III}\right|}_{y=d_{1}+d_{2}+d_{3}}={\left.\sigma_{12}^{IV}\right|}_{y=d_{1}+d_{2}+d_{3}}\;{\left.\sigma_{12}^{IV}\right|}_{y=d_{1}+d_{2}+d_{3}+d_{2}}={\left.\sigma_{12}^{V}\right|}_{y=d_{1}+d_{2}+d_{3}+d_{2}}\;;{\left.\sigma_{12}^{V}\right|}_{y=d_{1}+d_{2}+d_{3}+d_{2}+d_{1}}=0.$$

For isotropic media, mechanical stresses are defined as:

(5) $$\sigma_{12}^{II}=\sigma_{6}^{II}=2\mu_{2}u_{23}=\mu_{2}\left[\frac{\partial \xi_{y}}{\partial x}+\frac{\partial \xi_{x}}{\partial y}\right]=\mu_{2}\frac{\partial \xi_{x}}{\partial y}\left[\xi_{3}+\xi_{4}\right]=jz_{2}\omega \xi_{4m}e^{jk_{2}(y-d_{1})}-jz_{2}\omega \xi_{3m}e^{-jk_{2}\left(y-d_{1}\right)}.$$

Similar for other media:

(6) $$\sigma_{12}^{III}=\sigma_{6}^{III}=jz_{3}\omega \xi_{6m}e^{jk_{3}(y-d_{1}-d_{2})}-jz_{3}\omega \xi_{5m}e^{-jk_{3}(y-d_{1}-d_{2})}\;\;\sigma_{12}^{IV}=\sigma_{6}^{IV}=jz_{2}\omega \xi_{8m}e^{jk_{2}(y-d_{1}-d_{2}-d_{3})}-jz_{2}\omega \xi_{7m}e^{-jk_{2}\left(y-d_{1}-d_{2}-d_{3}\right)}.$$

In piezoelectric crystals, in addition to the elastic properties of the material, its piezoelectric properties are also taken into account:

(7) $$\sigma_{12}^{I}=\sigma_{6}^{I}=C_{66}u_{6}-e_{26}E_{2}$$

where $C_{66}$ is an effective modulus of elasticity of the piezoelectric material, and $e_{26}$ is the effective piezoelectric constant component. Since langasite belongs to symmetry class 32, $e_{26}=-e_{11}$. Thus:

(8) $$\sigma_{12}^{I}=\sigma_{6}^{I}=C_{66}u_{6}+e_{11}E_{2}$$

By definition, the deformation is described as:

(9) $$u_{6}=\frac{\partial \xi_{x}}{\partial y}=\frac{\partial }{\partial y}\left[\xi_{1}+\xi_{2}\right]=jk_{1}\left[\xi_{2}-\xi_{1}\right]$$

For langasite, taking into account its symmetry class, the component of the dielectric constant tensor of the medium is $\varepsilon_{22}=\varepsilon_{11}$, and the electrical induction is described as:

(10) $$D_{2}={e_{26}u_{6}+\varepsilon }_{22}E_{2}=-e_{11}u_{6}+\varepsilon_{11}E_{2}$$

The expression for $\sigma_{12}^{I}$ is obtained by integrating the electrical induction over the thickness of the plate and performing several simple mathematical transformations:

(11) $$\int_{0}^{d_{1}}D_{2}dy=\int_{0}^{d_{1}}-e_{11}u_{6}dy+\int_{0}^{d_{1}}\varepsilon_{11}E_{2}dy\;D_{2}d_{1}=-e_{11}\left[\xi_{2m}\left(e^{jk_{1}d_{1}}-1\right)+\xi_{1m}\left(e^{-jk_{1}d_{1}}-1\right)\right]+\varepsilon_{11}U_{0}\;D_{2}=-\frac{e_{11}}{d_{1}}\left[\xi_{2m}\left(e^{jk_{1}d_{1}}-1\right)+\xi_{1m}\left(e^{-jk_{1}d_{1}}-1\right)\right]+\frac{\varepsilon_{11}}{d_{1}}U_{0}\;E_{2}=-\frac{e_{11}}{d_{1}\varepsilon_{11}}\left[\xi_{2m}\left(e^{jk_{1}d_{1}}-1\right)+\xi_{1m}\left(e^{-jk_{1}d_{1}}-1\right)\right]+\frac{U_{0}}{d_{1}}+\frac{e_{11}}{\varepsilon_{11}}jk_{1}\left[\xi_{2}-\xi_{1}\right]\;\;\sigma_{12}^{I}=jC_{66}k_{1}\left[\xi_{2}-\xi_{1}\right]-\frac{e_{11}^{2}}{d_{1}\varepsilon_{11}}\left[\xi_{2m}\left(e^{jk_{1}d_{1}}-1\right)+\xi_{1m}\left(e^{-jk_{1}d_{1}}-1\right)\right]+\frac{{e_{11}U}_{0}}{d_{1}}+\frac{e_{11}^{2}}{\varepsilon_{11}}jk_{1}\left[\xi_{2}-\xi_{1}\right],$$

where $U_{0}=U_{0m}e^{j\omega t\;}$is the voltage applied to the radiating plate.

Similarly for the receiving plate:

(12) $$\sigma_{12}^{V}=jC_{66}k_{1}\left[\xi_{10}-\xi_{9}\right]-\frac{e_{11}^{2}}{d_{1}\varepsilon_{11}}\left[\xi_{10m}\left(e^{jk_{1}d_{1}}-1\right)+\xi_{9m}\left(e^{-jk_{1}d_{1}}-1\right)\right]+\frac{{e_{11}U}_{out}}{d_{1}}+\frac{e_{11}^{2}}{\varepsilon_{11}}jk_{1}\left[\xi_{10}-\xi_{9}\right].$$

To determine the output voltage $U_{out}$ on the load *Y*, it is necessary to supplement the system with one more equation:

(13) $$U_{out}=\frac{j\omega e_{11}S}{d_{1}(j\omega C_{p}+Y)}\left[\xi_{10m}\left(e^{jk_{1}d_{1}}-1\right)+\xi_{9m}\left(e^{-jk_{1}d_{1}}-1\right)\right],$$

where *S* is the area of the plate electrode, *C_p_* is the static capacitance of the plate, and *Y* is the complex conductivity of the load connected to the receiving plate.

As a result, the system of 11 linear algebraic equations with 11 unknowns (10 wave amplitudes and the output voltage $U_{out}$) is formed:

(14) $$M\left(f\right)\cdot X\left(f\right)=Q\left(f\right),$$

where **M**(*f*) is an 11 × 11 square coefficient matrix dependent on the frequency *f*; **X**(*f*) is the vector of unknowns; **Q**(*f*) is the vector of free terms determined by the input voltage $U_{0}$.

Solving this system for a given frequency range allows us to find the complex transfer function of the sensing element $U_{out}(f)/U_{0}$. Of particular interest is the analysis of the system’s frequency response $\left|U_{out}(f)/U_{0}\right|$, which contains information about the system’s resonant frequencies.

A special software was developed to numerically solve the problem. This program finds a solution to a system of linear equations for each frequency range and also calculates the system’s transmission coefficient for both standing and traveling waves. To solve the numerical task in traveling wave mode, the ξ_6_ wave must be excluded from the calculation (set its complex amplitude to zero) at the boundary *y* = *d*_1_ + *d*_2_. To ease the study of the influence of system parameters (layer thickness, elastic and piezoelectric properties of the layers and electrical load) a graphical interface based on the Python 3.14.4 tkinter library was created: the user can change parameters and observe the changes in the frequency response in a real-time mode.

Thus, the discussed mathematical model and its software implementation enable a comprehensive analysis and optimization of the proposed new gyroscope’s sensing element design.

### 2.3. Numerical Model

To verify the developed mathematical model, numerical studies were conducted, the results of which are presented as amplitude-frequency characteristics calculated using the designed software. The main results, demonstrating the influence of various parameters on the characteristics of the sensing element, are presented below.

**Figure 3:**
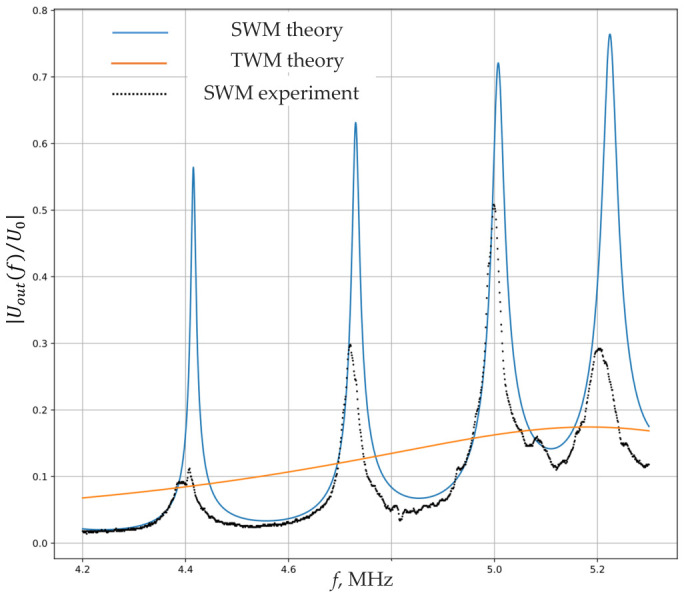
The frequency response of the layered plate-type vibrator for theoretical modeling and experimental research (SWM—the standing-wave mode; TWM—the traveling-wave mode).

Figure 3 shows a comparison of the frequency responses obtained from numerical simulation and measured on the experimental model. As shown in the graph, the resonant frequencies are in good agreement, which confirms the validity of the developed mathematical model. It is worth noting that the numerical model does not include parameters related to manufacturing inaccuracies (non-parallelism and surface roughness), nor does it take into account the frequency dependence of the attenuation coefficient, which is more pronounced on the experimental curve.

A comparison of the system’s transfer coefficients was also made when operating in traveling-wave and standing-wave modes. As seen from Figure 3, using the standing-wave mode increases the system’s transfer coefficient several times.

Numerical modeling revealed a significant influence of both contact layer parameters and electrical load parameters on the sensitive element characteristics. Thus, the influence of these parameters imposes certain constraints on both the manufacturing technology of the sensing element and the selection of optimal electrical matching circuits.

## 3. Experimental Setup

**Figure 4:**
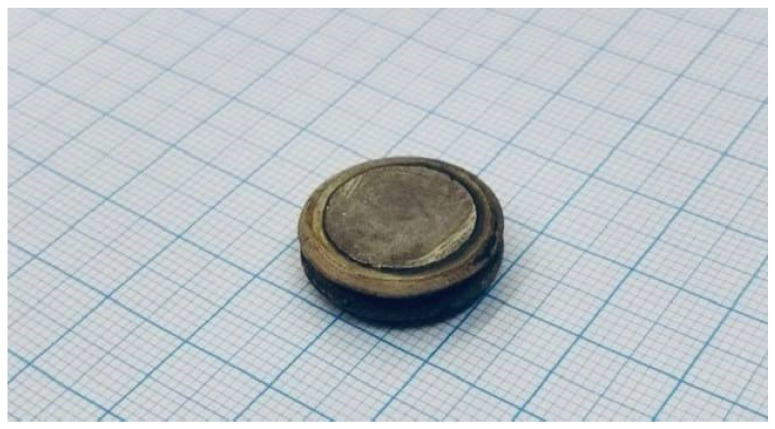
The sensing element of an angular velocity sensor on standing bulk acoustic waves.

The experimental prototype of an angular velocity sensor’s sensing element on standing waves is a disk with a diameter of 20 mm and a thickness of 5.8 mm, as shown in Figure 4.

The sensitive element is fixed to the mounting board, in which a hole is made for this purpose.

**Figure 5:**
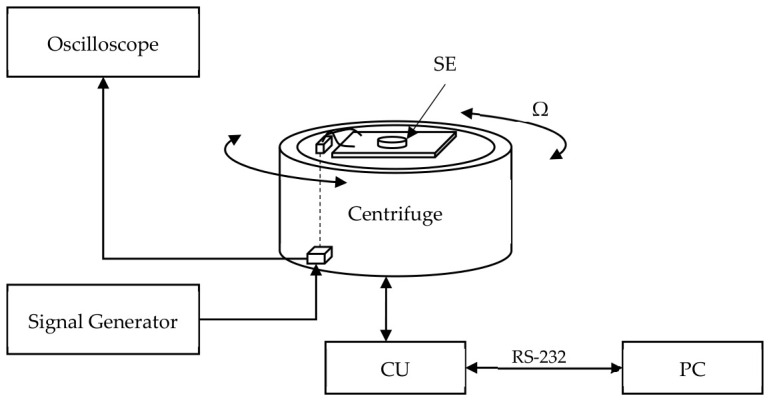
The block diagram of the experimental setup for the research of sensing element output characteristics under rotation.

The block diagram of the experimental setup is shown in Figure 5. The start, stop, and rotation velocity Ω of the centrifuge [21] are controlled by a personal computer (PC), which is connected to the centrifuge’s control unit (CU) via a COM port. The standing-wave sensing element (SE) is mounted on the centrifuge’s rotating stage. A signal generator AKИП 3402, АО «ПриCT», Russia, is connected to the transmitting plate via a sliding contact, generating a sinusoidal signal of a specified frequency. The receiving plate is connected via a sliding contact to the LeCroy WaveSurfer 24Xs oscilloscope, Teledyne Technologies Inc., U.S., which is used to measure changes in the output signal amplitude.

**Figure 6:**
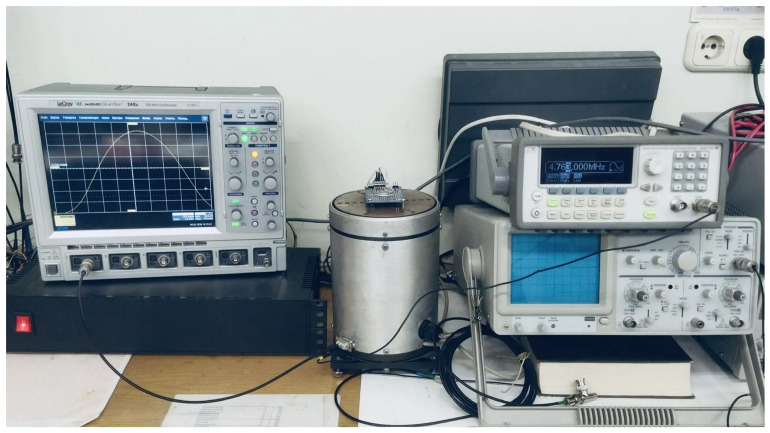
The assembled experimental setup for the research of sensing element output characteristics under rotation.

The assembled experimental setup is shown in Figure 6.

A continuous sine wave signal with a frequency of 4.763 MHz and an amplitude of 5 V is applied to the emitting plate. The input signal is determined by the frequency response of the sensitive element (see Figure 3) as the frequency near the amplitude resonance peak, which will allow obtaining the maximum gyroscopic efficiency and the maximum informative signal. The oscillatory circuit formed by the matching inductor and the receiving plate is directly connected to the oscilloscope. The oscilloscope screen displays a sinusoidal signal, which arises due to the imperfect orthogonal orientation of the emitting and receiving plates.

## 4. Experimental Results

**Figure 7:**
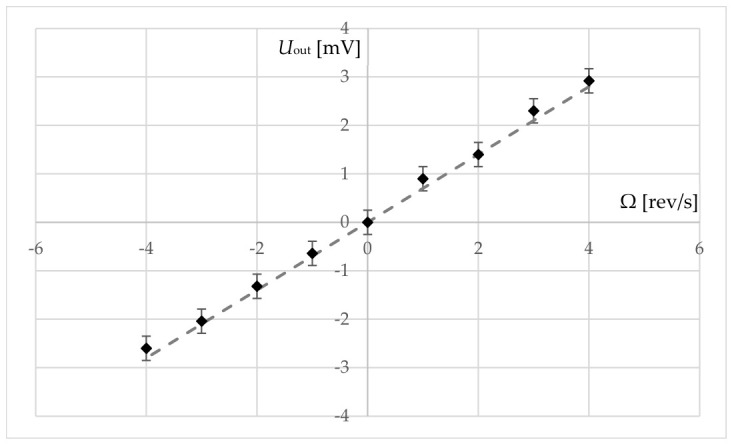
The informative signal dependence on the rotation velocity for a new standing acoustic wave sensing element.

As the centrifuge rotates, the signal amplitude on the oscilloscope screen begins to change relative to the static value. The dependence of the output voltage on rotation velocity is shown in Figure 7.

Measurements were performed at several rotation velocities. As shown in Figure 7, the output signal level changed from 0 mV at standstill to 2.8 mV at 4 rev/s. A linear fit to the experimental data yields a sensitivity of approximately 0.7 mV/(rev/s) with output characteristic linearity of about 99%.

It should be noted that raw sensitivity was obtained without dedicated signal processing circuitry, and by selecting optimal circuit design solutions the gyroscopic response can be amplified by one or two orders of magnitude, which makes this new type of shock-resistant ultrasonic gyroscopic sensor—a plate-type vibrator on standing acoustic waves—promising for further research.

In Table 1, a comparison of dimensions and sensitivity for various solid-state acoustic sensitive element types [19,20] is presented. It is seen that the new standing-wave concept allows minimizing the size while maintaining high conversion efficiency and achieving a competitive sensitivity level compared to previously reported concepts.

## 5. Discussion

1. As shown by the previous investigation, one of the critical challenges in designing acoustic wave gyroscopic sensors is to increase their sensitivity, given the typically small informative signal.
2. The new concept of shock-resistant ultrasonic gyroscopic sensor—a composite plate-type vibrator on standing waves is developed. The one-dimensional model of shear wave propagation in a five-layer vibrator structure (piezoelectric–adhesive–acoustic duct–adhesive–piezoelectric) demonstrated good agreement with experimental data on the position of resonant frequencies. Existing discrepancies in the resonance quality factors are explained by technological factors (surface roughness, non-parallelism of layers) not taken into account in the model and a simplified description of the frequency dependence of attenuation. While the proposed design theoretically offers high shock resistance due to the absence of moving parts, no direct shock or vibration tests were performed in this study; this remains a subject of future work.
3. Numerical modeling and experimental studies revealed, under the specific conditions of our model, a significant increase in the system’s transfer coefficient when operating in resonant standing-wave mode compared to the traveling wave mode. This allows for the minimizing of the size of the sensing element while maintaining high conversion efficiency, which is critical for the development of compact inertial sensors.
4. A novel type of shock-resistant ultrasonic gyroscopic sensor—a composite plate-type vibrator with orthogonally oriented piezoelectric transducers—has demonstrated a detectable gyroscopic response (0.7 mV/(rev/s)) without the use of additional analog signal processing tools. Results were obtained under laboratory conditions, indicating the feasibility of the proposed approach.
5. The obtained basic sensitivity (0.7 mV/(rev/s)) represents the “raw” signal without optimization of the circuit design. Further research is aimed at developing optimal methods for processing the received signal, which, according to preliminary estimation, will significantly increase the output sensitivity.
6. A promising direction for the development of a new standing-wave vibrator gyroscopic sensor is to convert the sensing element to a self-oscillation mode, in which the resonant system self-excites due to positive feedback. This mode ensures automatic tuning to the resonant frequency, minimizes the influence of parameter drift and potentially increases the stability of the output signal.
7. The subject of further research is the production of an industrial sample of a gyroscope using the developed sensitive element, as well as its testing to determine technical characteristics, including vibration and shock resistance.

## Figures and Tables

**Table 1:** A comparison of dimensions and sensitivity for various solid-state acoustic sensitive element concepts.

SE Concept	Length, mm	Sensitivity, mV/(rev/s)
Traveling-wave mode		
BAW polarization vector rotation type	17	0.5
Orthogonal-polarized BAW component type	70	0.1
Circular-polarized BAW type	150	0.34
Standing-wave mode	6	0.7

## Data Availability

The original contributions presented in this study are included in the article. Further inquiries can be directed to the corresponding author.

## Abbreviations

The following abbreviations are used in this manuscript:

MEMS	MicroElectroMechanical Sensor
OG	Optical Gyroscope
FOG	Fiber Optical Gyroscope
RLG	Ring Laser Gyroscope
WSG	Wave Solid-state Gyroscope
HRG	Hemispherical Resonator Gyroscope
ASG	Acoustic Solid-state Gyroscope
SAW	Surface Acoustic Wave
BAW	Bulk Acoustic Wave
IDT	InterDigital Transducer
SE	Sensitive Element
